# Research on Improving Moisture Resistance of Asphalt Mixture with Compounded Recycled Metallurgical Slag Powders

**DOI:** 10.3390/ma17143499

**Published:** 2024-07-15

**Authors:** Bo Gao, Haiqin Xu, Shaopeng Wu, Huan Wang, Xinkui Yang, Pengrui Chen

**Affiliations:** State Key Laboratory of Silicate Materials for Architectures, Wuhan University of Technology, Wuhan 430070, China

**Keywords:** moisture resistance, asphalt mixture, fillers, metallurgical slag

## Abstract

The utilization of steel slag as an alternative material in asphalt mixtures is considered the solution to the problem of the shortage of natural aggregates. However, asphalt mixtures with steel slag show susceptibility to damage caused by moisture, especially in powder form. Therefore, blast furnace slag powders were used to compound with steel slag powders as fillers to improve the moisture resistance of asphalt mixtures. The characteristics of the steel slag powders and blast furnace slag powders were investigated initially. Subsequently, the adhesion properties of the asphalt mastics with the powders to the aggregates were evaluated. Finally, the moisture resistances of the asphalt mixtures were identified. The results indicate that the steel slag powder exhibited a notable prevalence of surface pores, which had a more uniform size distribution. In contrast, the blast furnace slag powder exhibited a greater average pore size. The larger specific surface area of the steel slag powder was over 30% larger than that of the blast furnace slag powder, and the superior gelling activity of the blast furnace powder enhanced the adhesion property. Both the steel slag powder and blast furnace slag powder were found to enhance the adhesion properties of the asphalt mastics, while the effect of the steel slag powder was more pronounced, the maximum force difference of which exceeded 200 N. The antagonistic effect of the steel slag powder and blast furnace slag powder on the resistance of the adhesive interface to moisture damage was confirmed by the contact angle test. The incorporation of the blast furnace slag powder markedly enhanced the moisture resistances of the asphalt mixtures. The phenomenon of dynamic moisture damage to the asphalt mixtures was more pronounced under the multicycle times, obviously severer than that in a stable water environment. As the dynamic moisture cycles increased, the degree of destruction gradually approached a steady state.

## 1. Introduction

Asphalt materials are regarded as crucial components of contemporary pavement structures. Their exemplary service performances, encompassing comfort driving, minimal noise, optimal skid resistances, and straightforward maintenance, have rendered them a pervasive pavement material [1]. In Europe, the total road mileage is over 5.51 million kilometers, with over 90% of these roads paved with asphalt. These asphalt pavements support 80% of passenger transport and 70% of inland freight transport [2,3]. In China, over 90% of high-grade pavements are constructed with asphalt materials to complete the pavements [4]. The necessity of the construction of new roads to meet transport demand is increasing, while the task of maintaining existing roads is becoming increasingly onerous. In the period between 2000 and 2021, the total investment in road construction in Europe was approximately EUR 1880 billion, while EUR 1920 billion was spent on road maintenance [5]. The increasing engineering of new road construction and maintenance work on existing pavements directly results in a notable surge in the consumption of natural resources, including aggregates and asphalt binders [6,7]. Consequently, the advancement of greener and more sustainable materials for pavement construction and maintenance has emerged as a pivotal objective.

The utilization of bulk solid waste in road construction is currently regarded as a highly promising avenue for reducing the exploitation of natural resources and achieving green and sustainable development. Steel slag is a typical bulk solid waste, which is a by-product of the steelmaking process. The annual production of steel slag exceeds 120 million tons, representing 12% of crude steel production [8,9]. However, because of the poor stability and abrasiveness, it is a kind of solid waste that is difficult to be used in metallurgical slag, and its comprehensive utilization rate is less than 30% in China [10,11]. Although there are many studies and cases proving that steel slag can be used in asphalt mixtures, there are still some problems. Some previous studies have reported that steel slag has the advantages of high hardness, good angularity, and wear resistance [12,13,14]. These properties also give asphalt mixtures containing steel slag aggregates excellent slip resistance, high temperature stability, and fatigue durability [15,16,17]. However, some studies have reached different conclusions regarding its resistance to moisture. The results of some laboratory and field studies have shown that steel slag materials are sensitive to moisture damage, especially in powder form [18,19]. It was discovered that f-CaO in steel slag can be converted to CaCO_3_ by reacting with water and carbon dioxide (CO_2_) under moisture erosion. These enriched calcium carbonate deposits can lead to debonding of the steel slag–asphalt interface and, consequently, to damage, contributing to steel slag’s moisture sensitivity [20,21]. The specific surface area of powdered steel slag is greater, thereby increasing the likelihood of the swellable component being exposed to the moist environment, which, in turn, leads to damage [22,23]. Xiao et al. discovered that steel slag powders can be employed as a partial replacement for limestone filler, and the optimal substitution amount is 25% of the total volume of the filler [24]. Therefore, it is necessary to develop a filler material that can work with steel slag to avoid the defect of moisture sensitivity.

In consideration of the frequent occurrence of moisture damage to asphalt pavements, the most commonly used treatment method is adding agents that can prevent stripping. Liquid antistripping agents, such as the chemical surfactants of amines, have been demonstrated to have a good antistripping effect; however, they are susceptible to decomposition at high temperatures during the mixing process of over 180 °C, and their durability is highly questionable [25,26,27,28]. Inorganic antistripping agents, such as cement and lime, are widely used because of their good antistripping effect, heat resistance, and the easy availability of raw materials. Nevertheless, the considerable carbon footprint of materials such as cement has prompted the exploration of alternative materials as a promising avenue of research [29]. Blast furnace slag is a porous, amorphous silicate melt byproduct of the blast furnace ironmaking process. It has long been used as a supplementary cementitious material in cement concrete due to its similar chemical activity to that of cement, which also allows it to be used as a filler in asphalt concrete and to provide antispalling effects [30,31,32]. Concurrently, the annual output of blast furnace slag is in excess of 300 million tons, representing 35% of pig iron production, which is sufficient to meet the demands of engineering construction [33,34]. Because of its favorable gelling properties, blast furnace slag powder is frequently employed as a filler in cold mix asphalt mixtures. Its hydration reaction results in the formation of C-S-H gel, which has the potential to markedly enhance the engineering performance [35,36]. Lu et al. and Dulaimi et al. mixed blast furnace slag powder, fly ash, calcium carbide slag, and cement and found that this mixing system can enhance the interfacial adhesion performance, making it more suitable for pavements that are in harsh environments in terms of moisture and temperature [37,38]. Shaygan et al. explored the potential of blast furnace slag powder as a filler in microsurfacing mixtures, and their findings deemed acceptable the use of blast furnace slag powder as a substitute for up to 10% filler in a microsurfacing [39]. Huang et al. conducted an investigation into the potential use of blast furnace slag powder as a substitute for limestone filler in hot mix asphalt mixtures. Their findings indicate that the powder enhanced the elastic recovery and rutting resistance of the asphalt mastic, as determined through rheological tests and viscosity tests [40]. Ou et al. evaluated the possibility of replacing blast furnace slag powder as a filler using rheological and surface free energy theories. The results demonstrated that the addition of blast furnace slag powder improved the fatigue performance of asphalt mastic under strain loading, increased its fatigue life, and maintained its good performance under long-term loading [41]. Amin et al. employed it as a filler for dense-graded asphalt concrete, and the results of tests such as the wheel tracking test and four-point bending test demonstrated its suitability for use in asphalt pavements [42]. The above studies demonstrate that blast furnace slag powder has the potential to be employed as a filler, while its hydration and cementation products enhance the interfacial strength in harsh moisture environments, thereby improving the performance of asphalt mixtures below moisture erosion.

The incorporation of steel slag and blast furnace slag as fillers in asphalt concrete not only represents a resourceful use of solid waste and a reduction in the consumption of natural resources but also serves as an inorganic antistripping agent, thereby enhancing the resistance of asphalt pavements to water damage and facilitating their high-value utilization. This research proposed the use of compounded metallurgical slag powders as fillers in asphalt mixtures to improve the moisture resistance, and the characteristics of the metallurgical slags powder, adhesive properties of the asphalt mastics, and water resistance of the asphalt concrete were characterized. This proposition expands the utilization of metallurgical slags and eliminates the potential adverse effects of metallurgical slag on asphalt concrete. 

## 2. Materials and Methods

### 2.1. Raw Materials

The bitumen material selected for this research was AH-70 asphalt, obtained from Hubei Guochuang Hi-tech Material Co., Ltd. Basalt was employed as the aggregate in the asphalt concrete, which originated from Jingshan city in Hubei province, China. Table 1 presents the basic properties of the bitumen and basalt. In this research, limestone powder (LP), blast furnace slag powder (BP), and steel slag powder (SP) were utilized as fillers. Prior to their utilization, blast furnace slag and steel slag were crushed and sieved with a 0.075 mm mesh size. The properties of the powders are presented in Table 2. It can be observed that the properties of the selected materials all meet the requirements [43].

### 2.2. Sample Preparation

The asphalt and powders were selected for the preparation of the asphalt mastic. The Fillers–asphalt ratio was determined to be 30% by volume, in accordance with the established practice in the field and the findings of previous studies [44]. The preparation steps of asphalt mastics were carried out in accordance with the following procedure: (1) the asphalt and powders were initially preheated to 120 °C and 150 °C, respectively; (2) the asphalt was transferred to the container for an oil bath to maintain the temperature, after which it was premixed with a rotating speed of 500 rpm for 5 min; (3) the powders were added gradually with the same rotating speed, and until all powders had been added, the speed was increased to 1500 rpm for 10 min; (4) finally, the asphalt mastics were prepared and transferred to other containers for storage. Different formulations and ratios were used for the fillers, which are presented in Table 3. The AC-13 asphalt concrete used in this research was designed and prepared by the Marshall method [45]. The bitumen content was determined as 5%, and the targeted air content was 4.78%. The filler also adopted the above formulations and ratios. 

### 2.3. Experimental Methods

#### 2.3.1. Powders’ Characteristics

A QUANTA FEG 450 scanning electron microscope (SEM), made by FEI, Hillsboro, State of Oregon, USA, was used to characterize the surface appearance of the powders. The chemical compositions were characterized using the Zetium X-ray fluorescence (XRF) spectrometer, manufactured by Malvern Panalytical, Almelo, the Netherlands. The samples prepared by the tableting method were tested with a window rhodium target X-ray spectrographic tube of 4 kW. The phase distributions of the powders were characterized using a D8 Advance X-ray diffraction platform with 2θ from 20° to 80°. To obtain the particle size distributions of the powders, a Mastersizer 3000+, made by Malvern Panalytical, Netherlands, was used. Furthermore, an ASAP 2460, made by Micromeritics, Norcross, GA, USA, was used to detect the pore distributions ranging from 2 nm to 50 nm and the surface areas of the powders.

#### 2.3.2. Pull-Off Test

**Figure 1:**
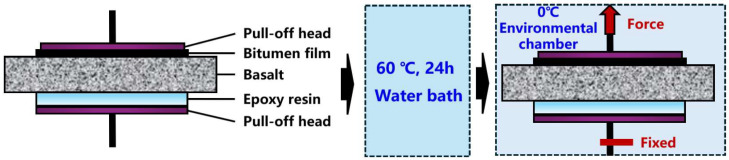
Procedures for the pull-off test.

The pull-off test between bitumen and aggregate would be conducted. The dimensions of the basalt block specimens were 20 mm × 20 mm × 5 mm. As illustrated in Figure 1, the undersurface of the block was affixed with a circular pushpin measuring 16 mm in diameter, embedded in an epoxy resin. The opposing face of the block was adhered with the pull-off head soaked in asphalt mastics. Subsequently, the pull-off system was maintained in a water bath at 60 °C for a period of 24 h in order to facilitate the completion of the moisture erosion process. Subsequently, the sample to be tested was prepared in accordance with the requisite specifications. The pull-off test was conducted with the bottom head fixed and the upper head loaded at 0 °C in the environmental chamber. The moisture resistance rate was employed to ascertain the adhesion properties between the asphalt mastics and basalt in a moist environment. This was achieved by utilizing Equation (1), as follows:

(1) $$MRT=\frac{F_{moistured}}{F_{virgin}}\times 100\%$$

where *MRT* is the moisture resistance rate; $F_{moistured}$ is the adhesive destructive force under moisture; and $F_{virgin}$ is the adhesive destructive force without moisture.

#### 2.3.3. Contact Angle Test and Surface Free Energy (SFE) Calculation

**Figure 2:**
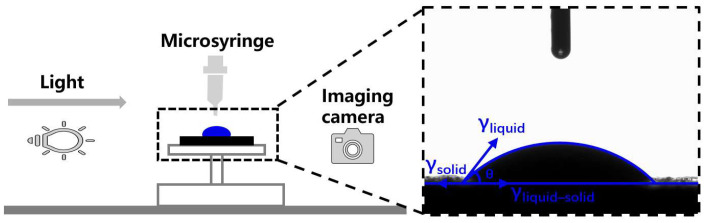
Contact angle test.

In this paper, the surface free energy theory was used to analyze the adhesion between the asphalt mastics and aggregates. The contact angles of the asphalt mastics and aggregates (limestone, basalt, andesite, and granite) were measured by the drop method, as illustrated in Figure 2. Dataphysics OCA20, DataPhysics Instruments GmbH, Filderstadt, Germany, was selected for the test. 

The contact angles of the aggregates were measured by the probe liquids, including distilled water, formamide, and glycol, while those of the asphalt mastics were measured using distilled water, formamide, and glycerol. Before a contact angle test, the asphalt mastics should be kept in a water bath of 60 °C for 24 h. The SFE parameters of the probe liquids used in this study are shown in Table 4. 

On the basis of the contact angle results, the parameters, such as surface energy, polar component, and dispersion component, of the aggregates and asphalt mastics can be obtained. The surface free energy, polar component, and dispersion component of the asphalt mastics can be calculated by Equations (2) and (3), as follows:

(2) $$\gamma_{L}=\gamma_{L}^{d}+\gamma_{L}^{P}$$

(3) $$\frac{\gamma_{L}(1+cos\theta )}{2}\frac{1}{\sqrt{\gamma_{L}^{d}}}=\sqrt{\gamma_{m}^{P}}\sqrt{\frac{\gamma_{L}^{P}}{\gamma_{L}^{d}}}+\sqrt{\gamma_{m}^{d}}$$

where $\gamma_{L}$ and $\gamma_{m}$ represent the SFE of the probe liquids and the samples, $\gamma_{L}^{d}$ and $\gamma_{L}^{P}$ represent the dispersive component and polarity component of the SFE of the probe liquids, and $\gamma_{m}^{d}$ and $\gamma_{m}^{P}$ represent the dispersive component and polarity component of the samples. Furthermore, the SFE parameters of the aggregates from our previous studies are shown in Table 5.

The energy required for separating the interface between the asphalt mastics and aggregates was determined as the adhesion work, which can show the level of difficulty for moisture to separate the asphalt mastic film from the aggregates. Because of the better adsorption of the aggregates in water, bitumen is continuously stripped from the surfaces of the aggregates. In the process, the asphalt–aggregate system separates into the following two systems: asphalt–water and aggregate-water. The work inside this process is determined as spalling work. The energy ratio (ER) was considered in the evaluation of the adhesion property of the asphalt mastics on the aggregates. The indexes are defined as Equations (4)–(6), as follows:

(4) $$W_{am}=\gamma_{a}+\gamma_{m}-\gamma_{am}=2\sqrt{\gamma_{a}^{d}\gamma_{m}^{d}}+2\sqrt{\gamma_{a}^{p}\gamma_{m}^{p}}$$

(5) $$W_{amw}=2\left(\sqrt{\gamma_{a}^{d}\gamma_{w}^{d}}+\sqrt{\gamma_{a}^{p}\gamma_{w}^{p}}+\sqrt{\gamma_{m}^{d}\gamma_{w}^{d}}+\sqrt{\gamma_{m}^{p}\gamma_{w}^{p}}-\sqrt{\gamma_{a}^{d}\gamma_{m}^{d}}-\sqrt{\gamma_{a}^{p}\gamma_{m}^{p}}\right)$$

(6) $$ER=\left|\frac{W_{am}}{W_{amw}}\right|\times 100\%$$

where $W_{am}$ refers to the adhesion work between the aggregates and asphalt mastics with water; $\gamma_{a}$, $\gamma_{m}$, and $\gamma_{am}$ refer to the SFEs of the aggregates, asphalt mastics, and aggregate–asphalt mastic system; $\gamma_{w}^{d}$ and $\gamma_{w}^{p}$ refer to the dispersive component and polarity component of the water; $W_{amw}$ refers to the spalling work; and ER refers to the energy ratio. 

#### 2.3.4. Moisture Resistance of Asphalt Mixture 

Moisture damage can cause asphalt film to wrap from aggregates gradually. In this research, the immersion Marshall test and freeze–thaw split test were chosen and carried out in accordance with ASTM D1075 and ASTM D4867 [46,47]. The residual Marshall stability (RMS) and tensile strength ratio (TSR) were used to evaluate the capacities of the asphalt mixtures to resist moisture damage, defined as the ratio of the Marshall stability before and after the water immersion Marshall test and the ratio of the indirect tensile strength before and after the freeze–thaw splitting test, respectively.

**Figure 3:**
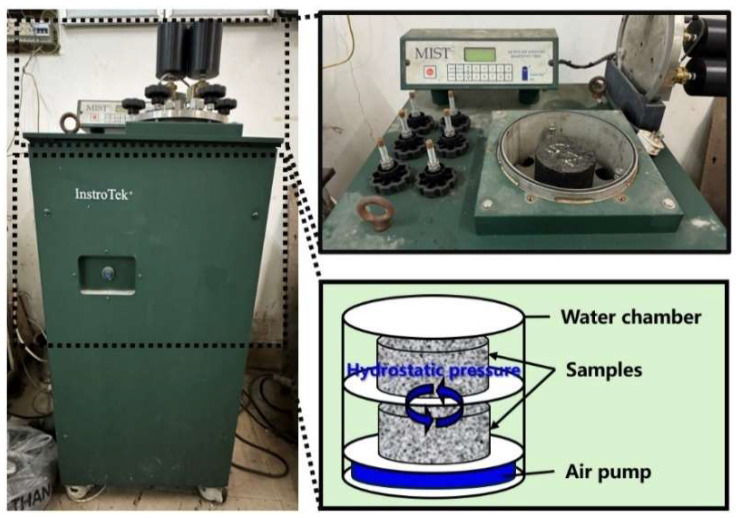
MIST device.

The MIST (Moisture Induced Sensitivity Tester) device, as shown in Figure 3, was produced to simulate dynamic moisture damage in the field. The air pocket inside the chamber can produce cyclical pressure and place it on the samples at a targeted temperature. The samples should be placed on the trays, filled with water, and conditioned using the MIST. After the samples are placed inside the MIST device, the temperature is raised to 60 °C, and the samples are required to remain in this environment for 20 h to simulate the chemical effect and potential adhesive failure. Then, the pressure is placed on the samples automatically by the expansion and contraction of the air pocket, driving moisture in and out of the voids of the samples repeatedly, similar to the pumping action of a driven car. A hydrodynamic cycle can be defined as the combination of the expansion and contraction of the air pocket. In this study, the samples were subjected to hydrodynamic pressure cycles, at 276 kPa, 2000 times, 3500 times, and 5000 times in order to simulate the mechanical effect of pore pressure experienced by pavement. Furthermore, the Marshall stability of the samples before and after the damage was evaluated, and the Marshall stability ratio (defined as the ratio of the Marshall stability before and after the dynamic moisture damage) was calculated to compare the dynamic moisture resistance. 

## 3. Results and Discussions

### 3.1. Material Characteristics

#### 3.1.1. Appearance, Pores, and Particle Size Distribution

**Figure 4:**
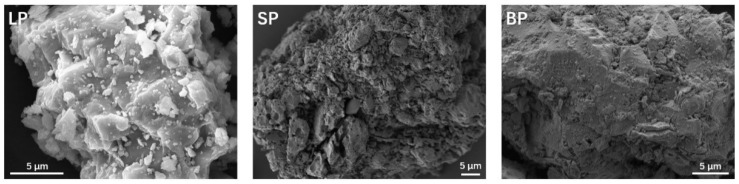
Micro-appearance of the powders.

Figure 4 shows the micro-appearance of limestone powders, steel slag powders, and blast furnace slag powders using the SEM device. It can be observed that the LP’s appearance was scaly and layered, the surface of which was smoothed but rugged due to the attached fine particles on the surface of the limestone powder. Furthermore, the sizes of the particles differed obviously. The appearance of the SP was completely different, whose surface should be defined as severely rough. A micro-gully can easily be observed, and abundant pores of multiple sizes can also be found, which contributed to the roughness of the surface of the SP. Gullies and pores increase the specific surface area of steel slag powders and improve the adhesion between steel slag powders and asphalt; however, they also increase the risk of moisture damage at the interface once cracks and stripping occur. The surface appearance of the BP was uneven, and its surface also had fine particles attached, but their numbers were fewer, and their particle sizes were smaller and more uniform. The surface roughness of the BP was between the SP and LP. There were some unique textures on the surface of the BP in addition to the attached particles. These particles and textures enhance the adhesion property, and the lack of obvious gullies and pores make invasion into the interior of the BP harder, unlike the SP, when eroded by moisture, which helps improve its moisture resistance.

**Figure 5:**
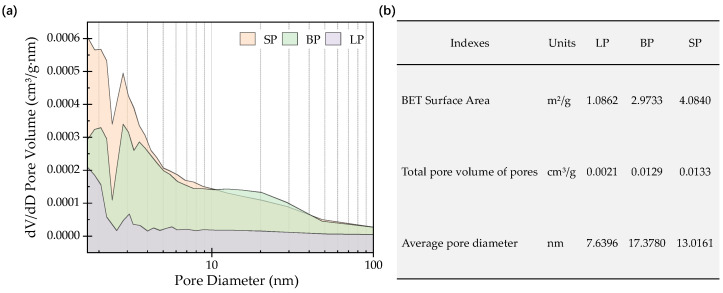
Pores distribution of the powders: (**a**) dV/dD pore volume distribution; (**b**) pores characteristics index.

Figure 5a indicates the pore distribution of the powders. The area enclosed by the curve and the coordinate axis is the sum of the hole volumes. It can be found that the SP had the largest pore volume, followed by the BP, and the smallest belonged to the LP. The value of the vertical axis qualitatively represents the number of holes. In the pore size range of 2–10 nm, the SP had the largest number of pores, and in the pore size range of 10–100 nm, the BP had the largest number of pores and the largest volume. Figure 5b shows the pore characteristics, including the BET surface area, total pore volume of the pores, and average pore diameter. The SP had the largest surface area, which can contribute to increasing the adhesion property with asphalt. Combined with the results of the average pore size, it can be seen that the pores in the BP were larger but fewer, and the pores in the SP were more balanced and numerous.

**Figure 6:**
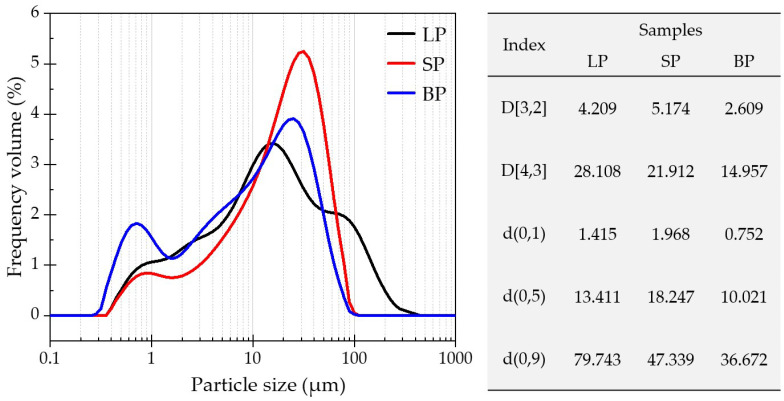
Particle size distributions of the powders.

Figure 6 presents the particle size distribution of the LP, SP, and BP. The frequency distribution–particle size curves show that the main concentration ranges of the LP, SP, and BP were around 10–20 μm, 20–40 μm, and 10–30 μm, respectively. The BP had another unique concentration in the range of 0.5–0.8 μm. It can also be found that the peak of the SP was larger than for the LP and BP, which indicates that the particle size distribution in the main concentration range of the SP was more concentrated while that of the LP was more uniform. D[3,2] and D[4,3] are the surface-weighted mean diameter and volume-weighted mean diameter, respectively. d(0,1), d(0,5), and d(0,9) are the accumulated values under the particle size volume distributions, which indicate that particles smaller than the particle size values in the samples accounted for 10%, 50%, and 90% of the total sample volumes, respectively. The difference of D[3,2] and D[4,3] reflects the concentrations of the particle size distributions, as follows: a larger difference results in a wider particle size distribution. It can be found that the differences for the LP, SP, and BP were 23.899, 16.738, and 12.348, respectively, which demonstrates that in the whole particle size range, the particle size distribution of the BP was the most concentrated and that of the LP the most uniform. The difference of D[4,3] and d(0,5) reflects the symmetries of the particle size distributions, as follows: a larger difference results in severe asymmetry. The differences of the LP, SP, and BP were 14.697, 3.665, and 4.936, respectively, indicating the better symmetries of the distributions of the SP and BP. Overall, the particle size distributions of the SP and BP were concentrated and symmetrical while that of the LP more uniform but asymmetrical. Furthermore, the smaller d(0,9) diameter values for the SP and BP would produce the desired compatibility with asphalt.

#### 3.1.2. Chemical Compositions and Phase Distributions

The XRF results of the powders are presented in Table 6. It can be found that the Ca element was the main component of the powders. The BP had certain amounts of the Si and Al elements, and the SP had certain amounts of the Si, Fe, Mg, and Al elements. The elements in the BP and SP contributed to the formation of the different crystals in the slag. The Si and Al elements in the BP make it easy for aluminosilicate to form, while the Si, Fe, Mg, and Al elements in the SP contribute to the formation of calcium iron oxide, aluminosilicate, and silicate. Furthermore, the ignition loss of the LP was found to be obviously larger, which is caused by the decomposition of CaCO_3_ during the period in which CaO and CO_2_ would be produced.

**Figure 7:**
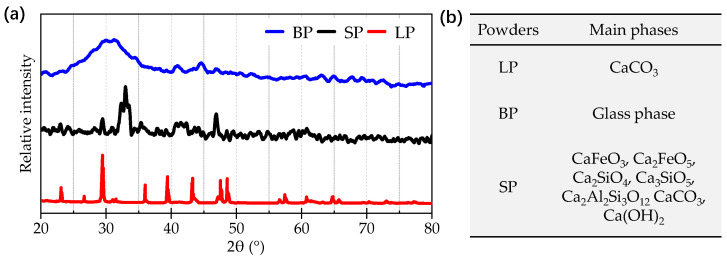
XRD results of the powders: (**a**) relative intensity; (**b**) main phases.

Figure 7a demonstrates the XRD results of the powders. It can be obviously found that the miscellaneous peaks are hard to observe in the curve of the LP; meanwhile, the peaks of the LF are sharper with a stronger relative intensity than those of the SP and BP. The curve of the BP shows a broad bump from 2θ = 25° to 2θ = 35°, which is composited by the glass phase that is manufactured by the rapid cooling of the slag produced by the blast furnace ironmaking. The curve of the SP consists of numerous miscellaneous peaks and some obvious main peaks. Figure 7b lists the main phases of the powders, indicating that the LP was completely composed of CaCO_3_, which is consistent with the fact that the LP was ground from calcite. The phase distributions of the SP were more complex. It can be found that the SP was composed of calcium iron oxide, aluminosilicate, silicate, and weathering products, which include CaFeO_3_, Ca_2_FeO_5_, Ca_2_SiO_4_, Ca_3_SiO_5_, Ca_2_Al_2_Si_3_O_12_, CaCO_3_, and Ca(OH)_2_. The steel slag is in a molten state before being discharged, and the CaO-Al_2_O_3_-SiO_2_-FeO slag system will form various crystals. The existence of CaCO_3_ and Ca(OH)_2_ is caused by the reaction between f-CaO and water in the environment. Ca(OH)_2_ is the intermediate product of the reaction. A glass phase is detected in the curve of the BP, which is the CaO-Al_2_O_3_-SiO_2_ slag system. 

### 3.2. Adhesion Property

**Figure 8:**
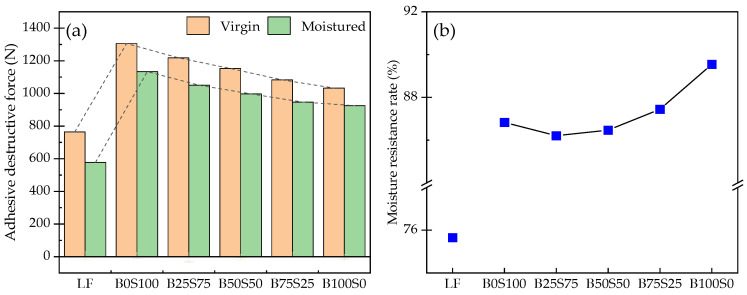
Results of the pull-off tests: (**a**) adhesive destructive force; (**b**) moisture resistance rate.

Figure 8 shows the results of the pull-off tests with the asphalt mastics. In Figure 8a, the adhesive destructive forces before and after the moisture damage are shown. The adhesive destructive forces of the asphalt mastics after the moisture damage were all less than those before the damage, indicating the deterioration of the adhesion property by the moisture damage. It can be found that the adhesive destructive forces of the LF asphalt mastic before and after the moisture damage were all less than those of the BP–SP asphalt mastics, indicating that the adhesion property of the LP asphalt mastic with basalt was weaker than those of the BP–SP asphalt mastics. With the increment in the proportion of the BP powders, the virgin and moisture adhesive destructive forces showed a clearly decreasing tendency, which reveals the better contribution of the SP to the adhesion property of the asphalt mastics with the aggregate. The tendency of the moisture force was more gradual than the virgin force, showing the homogenization of the moisture damage. Figure 8b shows the moisture resistance rates of the asphalt mastics. With the increase in the BP powder’s content, the moisture resistance rate displayed the tendency of declining at the beginning and rising later. The moisture resistance rates of the B100S0 asphalt mastics increased to greater than that of the B0S100 asphalt mastics. These results demonstrate the antagonistic effects between the SP and BP on the adhesion property. When a small amount of the SP was replaced, the adhesion property was damaged quickly, but the added BP failed to make up for this deficiency, until more of the SP was replaced, which finally increased it. 

**Figure 9:**
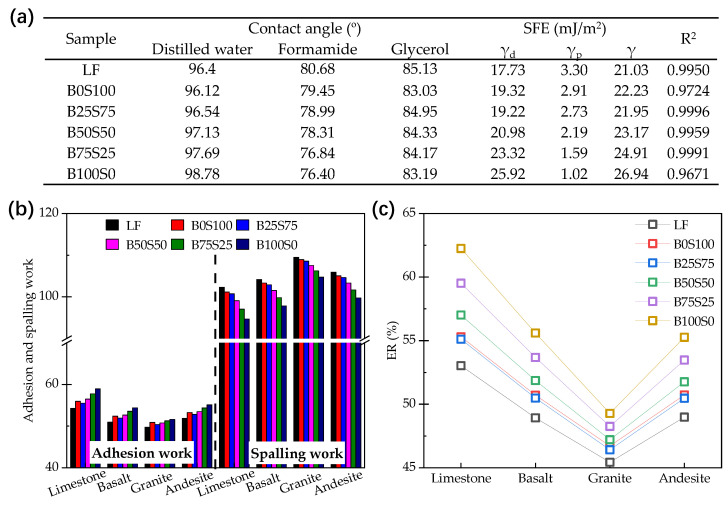
Evaluation of the adhesion properties of the asphalt mastics: (**a**) contact angle results and SFE parameters; (**b**) adhesion work and spalling work; (**c**) ER.

Figure 9a shows the contact angle results and the SFE parameters of the asphalt mastics. It can be found that the contact angles on the asphalt mastics were larger than 90°, which indicates that the addition of the powders would not change the hydrophobic property of asphalt. In accordance with the results of the SFE for the asphalt mastics and Equations (4)–(6), the adhesion work, spalling work, and ER were obtained, as shown in Figure 9b,c. The adhesion work between asphalt and aggregate is the energy required to separate under dry conditions. The higher the adhesion work, the more energy that is required to separate the two phases. The spalling work is the energy released when moisture replaces the asphalt film on the surface of the aggregate. The greater the spalling work, the more energy that is released in the process of changing from a two-phase to a three-phase system. It is clear that the adhesion effect with limestone was the most effective, followed by andesite, basalt, and granite, which is consistent with practical experience. This is also evidenced from the spalling work, which resulted in limestone, basalt, andesite, and granite, in descending order, suggesting that the asphalt–granite interface was most susceptible to moisture damage. It can be also found in Figure 9b that with the increment in the BP’s content, the adhesion work decreased slightly and then increased rapidly; meanwhile, the spalling work decreased continuously. The results show that the moisture damage to the BP would make the asphalt mastic adhere more to the aggregates. In the erosion process, the components in the BP and SP would react with water and generate C-S-H gels and CaCO_3_, respectively, the former of which would make a greater contribution to the adhesion. The results in Figure 9c also support the idea that the BP can increase the adhesion property of the asphalt mastics with the aggregate after moisture damage. 

### 3.3. Moisture Susceptibility

**Figure 10:**
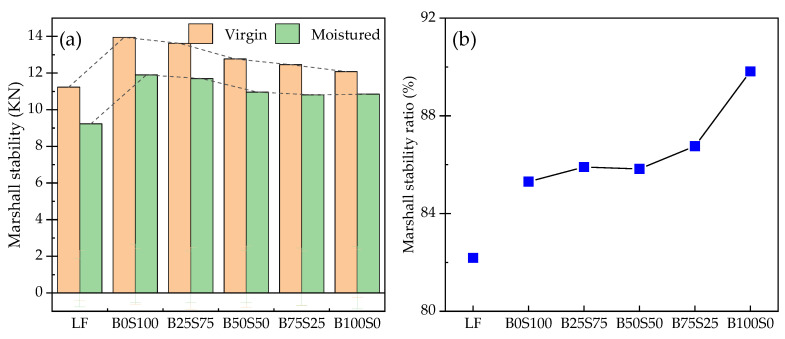
Results of the immersion Marshall test: (**a**) Marshall stability; (**b**) RMS.

Figure 10 shows the results of the immersion Marshall test. As can be seen from Figure 10a, the RMS of the Marshall specimens after the powders’ addition was higher than 80%, which is higher than the specification’s requirements. Under dry conditions, the Marshall stability of the LP as a filler was obviously low, while the Marshall stability of the 100%SP as a filler was the highest. As the BP content increased, the Marshall stability of the sample gradually decreased, but after the addition of 50% BP, the decrease tended to become stable. This indicates that the SP is more suitable as a filler under dry conditions. After the water immersion treatment, the Marshall stability of the samples decreased. When the BP content reached 50%, the Marshall stability of the specimen remained basically unchanged. By comparing the RMS of the specimens, it can be seen that when the content of BP was less than 50%, the residual stability of the specimens was in a state of fluctuation, and the SP and BP were antagonistic at this time. On the one hand, the SP and BP can improve the adhesion performance, but the residual f-CaO in the SP would react with water, which may expand the residual volume, destroy the stability of the Marshall specimen, and offset the improvement in the adhesion performance. When the BP content is above 50%, the effect of increasing the BP is advantageous and the residual stability will gradually increase.

**Figure 11:**
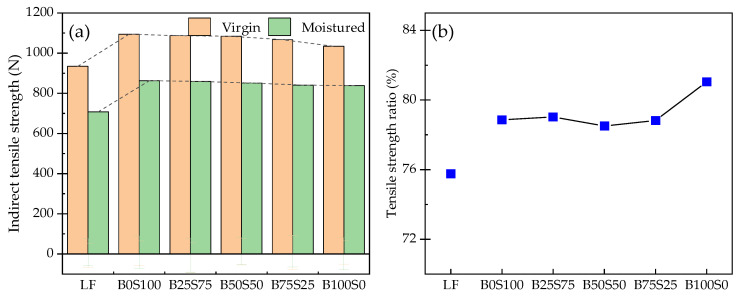
Results of the freeze–thaw split tests: (**a**) indirect tensile strength; (**b**) TSR.

Figure 11 shows the results of the freeze–thaw split tests. The regularity of the freeze–thaw splitting test results is similar to that of the Marshall stability test results. The addition of steel slag powder and blast furnace slag powder can improve the TSR value of the Marshall specimen. Under the same dosage, the TSR value of the SP group was the highest. Under the drying condition, with the decrease in the steel slag powder content, the indirect tensile strength of the specimen gradually decreased, but the indirect tensile strength of the specimen changed little after the freeze–thaw cycle. After the freeze–thaw cycle, the TSR value of the BP group was the highest under the same dosage, which shows that the steel slag powder had the best effect on the water damage resistance of the concrete under the same replacement dosage.

### 3.4. Dynamic Moisture Damage Resistance

**Figure 12:**
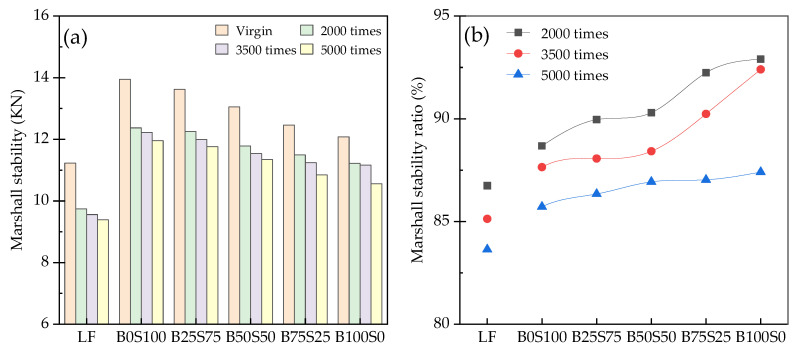
Moisture resistances of the asphalt mixtures after the MIST conditioning: (**a**) Marshall stabilities; (**b**) Marshall stability ratios.

Figure 12 shows the moisture resistance of the asphalt mixtures after the MIST conditioning. In Figure 12a, the Marshall stabilities of the asphalt mixtures after the MIST conditioning with different cycles are shown. The Marshall stabilities of the asphalt mixtures after the MIST conditioning were obviously lower than those of the virgin samples. After the MIST conditioning, the Marshall stability of the B0S100 was still the highest, but the difference between the B0S100 and the other samples was obviously reduced. More dynamic cycles lead to a greater decrease in the Marshall stability. The average decreases after 5000 times for the LF, B0S100, B25S75, B50S50, B75S25, and B100S0 compared to the virgin samples were found to be 16.4%, 14.3%, 13.7%, 13.1%, 13.0%, and 12.6%, respectively. Figure 12b shows the Marshall stability ratios of the asphalt mixtures after the MIST treatment. The lower value for the LF indicates a higher degree of moisture damage. Increasing the BP content is helpful for the asphalt mixtures in resisting dynamic water damage. However, as the number of dynamic water cycles increases, the effect of this increase is gradually reduced.

## 4. Conclusions

The improvements in the moisture resistances of the asphalt mixtures with compounded recycled metallurgical slag powders were investigated in this research. The above analysis and discussion led to the following conclusions:

Steel slag powder is characterized by a high specific surface area, which is a consequence of its porous structure and uniform particle size. In contrast, blast furnace slag powder exhibits a lower specific surface area but a larger average particle size. The adhesion property of steel slag powder is enhanced by its larger specific surface area, which is over 30% larger than blast furnace slag powders while that of blast furnace slag powders is enhanced by the gelling activity.

Both steel slag powder and blast furnace slag powder have the capacity to enhance the adhesion performance of asphalt mastics. However, the effect of steel slag powder is more pronounced, the maximum force difference of which exceeds 200 N. The antagonistic effect of steel slag powder and blast furnace slag powder on the ability of the adhesion interface to resist moisture damage was observed through the trend of the moisture resistance first decreasing and then increasing.

The invasion of moisture will result in the deterioration of the mechanical properties of the asphalt mixture, especially for the samples with LF, some indicators of which dropped by more than 20%. The incorporation of blast furnace slag powder serves to enhance the resistance of the asphalt mixture to such invasion. Concurrently, the antagonistic effect of steel slag powder and blast furnace slag powder on water is also evident in the scale of the asphalt mixture. The damage caused to asphalt mixtures by dynamic moisture is more pronounced. As the circulation time increases, the damage trend will tend to become more stable, and with the damage level gradually approaching a steady state, the difference in the indexes over time will be less than 5%. 

In conclusion, the incorporation of steel slag powder has been demonstrated to enhance the intrinsic mechanical properties of asphalt mixtures. The incorporation of blast furnace slag powder was shown to enhance the resistance of asphalt mixtures to moisture damage, although this may result in an antagonistic effect with steel slag powder. Therefore, it is essential to exercise caution when controlling the dosage in practical engineering applications. The combined addition of steel slag powder and blast furnace slag powder can result in a synergistic effect on asphalt mixture, leading to enhanced mechanical properties and heightened moisture resistance.

## Figures and Tables

**Table 1:** Basic properties of bitumen and aggregate.

Materials	Properties	Values	Requirements
AH-70 asphalt	Penetration (25 °C, 0.1 mm)	65.9	60~80
	Ductility (15 °C, cm)	>100	≥40
	Softening point (°C)	46.1	43
	Density (g/cm^3^)	1.032	-
Basalt	Coarse aggregates density (g/cm^3^)	2.89	≥2.5
	Fine aggregates density (g/cm^3^)	2.87	≥2.6
	Los Angeles abrasion	16.5	≤28
	Crush values	14.9	≤26

**Table 2:** Properties of the powders.

Properties	Powders			Requirements
	LP	BP	SP	
Density (g/cm^3^)	2.691	2.912	3.787	≥2.5
Hydrophilic coefficient	0.716	0.742	0.696	<1.0
Water content (%)	0.423	0.554	0.783	≤1.0

**Table 3:** Formulas and abbreviations of the asphalt mastics.

Formula	Abbreviation					
	LF	B0S100	B25S75	B50S50	B75S25	B100S0
Fillers–asphalt ratio	30%					
LP	100%	/	/	/	/	/
BP	/	/	25%	50%	75%	100%
SP	/	100%	75%	50%	25%	0%

**Table 4:** SFE parameters of the probe liquids.

	Distilled Water	Formamide	Glycerol	Glycol
$\gamma_{L}^{d}$ ^1^ (mJ/m^2^)	21.8	39.0	34.0	29.3
$\gamma_{L}^{P}$ ^2^ (mJ/m^2^)	51.0	19.0	30.0	19.0
$\gamma_{L}$ ^3^ (mJ/m^2^)	72.8	58.0	64.0	48.3

^1^ $\gamma_{L}^{d}$ is the dispersion component of the probe liquids. ^2^ $\gamma_{L}^{P}$ is the polar component of the probe liquids. ^3^ $\gamma_{L}$ is the surface free energy of the probe liquids.

**Table 5:** Contact angle results and SFE parameters of the aggregates.

Sample	Contact Angle (°)			SFE (mJ/m^2^)			R^2^
	Distilled Water	Formamide	Glycol	$\gamma_{a}^{d}$ ^1^	$\gamma_{a}^{p}$ ^2^	$\gamma_{a}$ ^3^	
Limestone	75.2	58.1	39.9	26.93	9.16	36.09	0.9947
Basalt	76.3	62.2	46.8	22.07	10.55	32.62	0.9987
Granite	72.8	60.7	48.1	18.65	14.33	32.98	0.9962
Andesite	74.1	59.5	44.6	22.45	11.61	34.06	0.9997

^1^ $\gamma_{a}^{d}$ is the dispersion component of the aggregates. ^2^ $\gamma_{a}^{p}$ is the polar component of the aggregates. ^3^ $\gamma_{a}$ is the surface free energy of the aggregates.

**Table 6:** Average chemical compositions of the powders.

Compound		CaO	Fe_2_O_3_	SiO_2_	MgO	Al_2_O_3_	Others
Proportion (wt%)	LP	55.54	1.77	1.57	0.53	0.17	40.42
	BP	40.42	0.48	32.22	7.39	14.99	4.50
	SP	33.27	16.73	13.95	13.55	12.29	10.21

## Data Availability

The data presented in this study are available on request from the corresponding author.
